# Effect of intra-socket application of hyaluronic acid gel on soft and hard tissue healing following impacted mandibular third molars extraction (a randomized controlled clinical trial)

**DOI:** 10.1186/s12903-025-05530-4

**Published:** 2025-02-10

**Authors:** Omnia Elkady, Osama Sweedan, Tasneem Amer

**Affiliations:** Faculty of Dentistry Alexandria University, Alexandria, Egypt

**Keywords:** Intra-socket, Hyaluronic acid gel, Tissue healing, Mandibular third molars

## Abstract

**Background:**

One of the most frequent dental operations is the surgical extraction of an impacted third molar. The study aimed to evaluate the impact of the intra-socket application of 0.8% Hyaluronic Acid (HA) gel on hard & soft tissue healing after surgical removal of the impacted 3rd molar.

**Objective:**

**Materials and methods:**

This randomized controlled clinical study included thirty patients aged from 21–36 years who were scheduled for surgical removal of impacted mandibular third molar. 0.8% hyaluronic acid gel (HA group) was applied immediately after surgery in the intra-socket of 15 patients, and nothing (No HA group) was applied to the socket of the other 15 patients. Soft tissue healing was assessed after extraction on the third, seventh, and fourteenth day. Bone healing was assessed 2 months post-extraction by measuring bone density and socket length from cone beam computerized tomography.

**Results:**

The soft tissue healing index was significantly better (very good or excellent) in the HA group compared with no HA group after fourteen days postoperative (*p* < .001). Also, the percentage increase of bone density in the HA group was statistically significantly higher after two months than in the no HA group ((*p* < .001).

**Conclusion:**

Hyaluronic acid 0.8% gel application improves soft tissue healing and bone density healing following surgical extraction of the mandibular third molar. It could be considered a valuable material for improving bone healing and soft tissue.

**Trial registration:**

The trial is retrospectively registered at the Pan African Clinical Trial Registry with the identification number for the registry PACTR202407576478340 on 30/07/2024.

## Background

Surgical removal of the impacted third molar is one of the most common surgical events [1]. This procedure often leads to postoperative pain, inflammation within 24 to 72 h, and trismus, all of which contribute to significant patient discomfort [2]. Additionally, the extraction of the third molar can impact the periodontal health of the lower second molar [2–4]. Moreover, the type of surgical wound healing and regeneration is closely linked to the levels of pain and inflammation experienced postoperatively [5].

Successful soft tissue healing relies on the coordinated interaction of various tissues and cell types [6, 7]. The healing process involves several stages: inflammation, coagulation, tissue resolution, and replacement. During the coagulation phase, platelets release growth factors, chemokines, and matrix components [8]. Various systemic drugs, preservative materials, natural substances, and irrigation techniques have been employed to mitigate complications and accelerate the healing process [9].

Hyaluronic acid, also known as hyaluronan or sodium hyaluronate, is a large molecule composed of repeating non-sulfated disaccharide units of D-glucuronic acid and acetyl glucosamine, forming a high molecular weight glycosaminoglycan structure [10]. Hyaluronic Acid (HA) is a biologically significant substance present in various tissues throughout the body, including synovial fluid, cartilage, skin, eyes, tendons, and most body fluids. It also acts as a key component of the extracellular matrix [11, 12].

Hyaluronic acid has been shown to significantly accelerate soft tissue healing by promoting the formation of early granulation tissue, supporting re-epithelialization and angiogenesis, and reducing damage and inflammation. It is also effective in minimizing postoperative swelling and inflammation [13]. Due to its growing use in dentistry, hyaluronic acid has been suggested to impact periodontal regeneration [14] positively. Furthermore, hyaluronic acid is crucial for bone regeneration as it promotes the proliferation, adhesion, and migration of undifferentiated mesenchymal cells into osteoblastic cells [13, 15].

The objective of the study was to evaluate the effect of applying 0.8% hyaluronic acid gel directly into the socket on the healing of soft tissues following the extraction of an impacted third molar.

## Methods

This study was conducted in full accordance with the ethical principles, including the World Medical Association Declaration of Helsinki, and authorized by the Institutional Review Board at the Faculty of Dentistry, Alexandria University, Egypt, in 22 January 2023 (IORG0008839). Written informed consent was obtained before enrollement. This study also adheres to the CONSORT guidelines 2010. The study started February 2023 and ended July 2024.

In this parallel randomized controlled clinical trial, thirty patients treated at the Outpatient Clinics of the Maxillofacial and Oral Surgery Department at the Faculty of Dentistry, Alexandria University, Egypt were enrolled. The PICO question was: Do patients present with impacted mandibular third molars? The research utilized a randomized design with balanced allocation ratio (1:1) comparing the intra-socket application of 0.8% hyaluronic acid gel versus no hyaluronic acid gel after surgery. The findings indicated an acceleration in the healing of both soft and hard tissues.

### Sample size evaluation

The minimal sample size is calculated based on a previous study aimed to evaluate the efficacy of 0.2% hyaluronic acid gel and 0.01% hyaluronic acid (HA) spray in the healing of extraction wounds using the ruler and digital planimetry method [16]. Ibraheem et al. (2022) [16], concluded that hyaluronic acid offers a beneficial effect in early post-operative healing after extraction. The sample size was calculated to detect the difference in soft and hard tissue healing. Based on Ibraheem et al. (2022) [16] results, adopting a power of 80% (β = 0.20) to difference in tissue healing, and a level of significance of 5% (α error accepted = 0.05), the minimum required sample size was found to be 12 patients per group (number of groups = 2) (Total sample size = 24 patients) [17, 18]. After adjustment for a dropout rate of 20%, the sample size was increased to 15 patients per group (number of groups = 2) (Total sample size = 30 patients) [19].

### Eligibility criteria

*Age:* 20 to 40 years.

*Sex:* Both.

*Inclusion criteria:* (mesioangular impacted mandibular third molar class II) according to Pell and Gregory classification.

*Exclusion criteria:* Any conditions affecting bone healing, bleeding disorders, acute infections at the surgical site or purulent discharge, those who smoke more than twenty-five cigarettes per day, alcoholics, and women using contraceptives or corticosteroids may experience complications affecting the healing phase after surgery.

### Sample allocation and concealment

The eligible patients were allocated to two balanced groups using simple random allocation and allocated table were generated using computer-generated random numbers.

The random allocation sequence, the enrolled of participants and participants assignment to interventions were carried out by third party (specialized statistics team). Participants and Outcome assessors were blinded to allocation.

HA Group: Received the application of hyaluronic acid (HA) gel in the socket of 15 patients following the extraction of impacted mandibular third molars.

No HA Group: Did not receive HA gel application in the socket.

No changes in the methodology after trial commencement.

### Materials

- 0.8% hyaluronic acid gel. Gengigel® Prof Syringes, Ricerfarma, Italy

### Intervention

#### Pre-surgical assessment

The patient's medical histories, including name, age, gender, occupation, address, and general health, were recorded. A clinical examination was assessed for suppuration, discharge, or swelling, while a radiographic examination was performed to evaluate the eruption of the third molar.

#### Preoperative patient preparation

Before the procedure, all patients in both groups underwent oral preparation by rinsing their mouths with an antiseptic mouthwash.

### Surgical phase

**Fig. 1:**
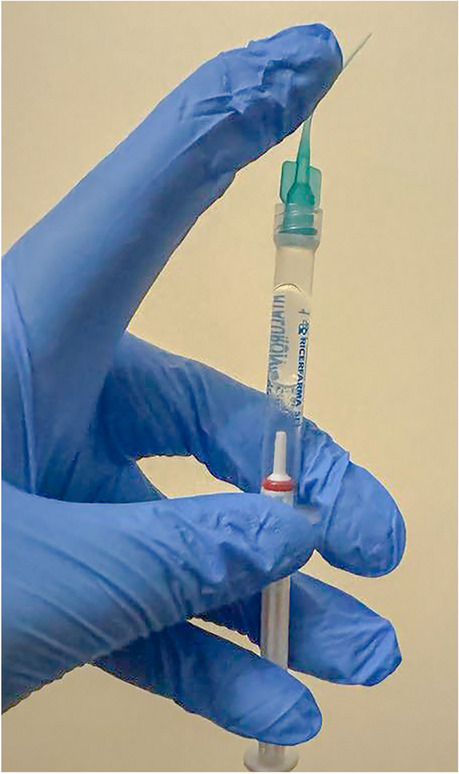
Syringe of 0.8% hyaluronic acid gel with a sterile disposable plastic needle

**Fig. 2:**
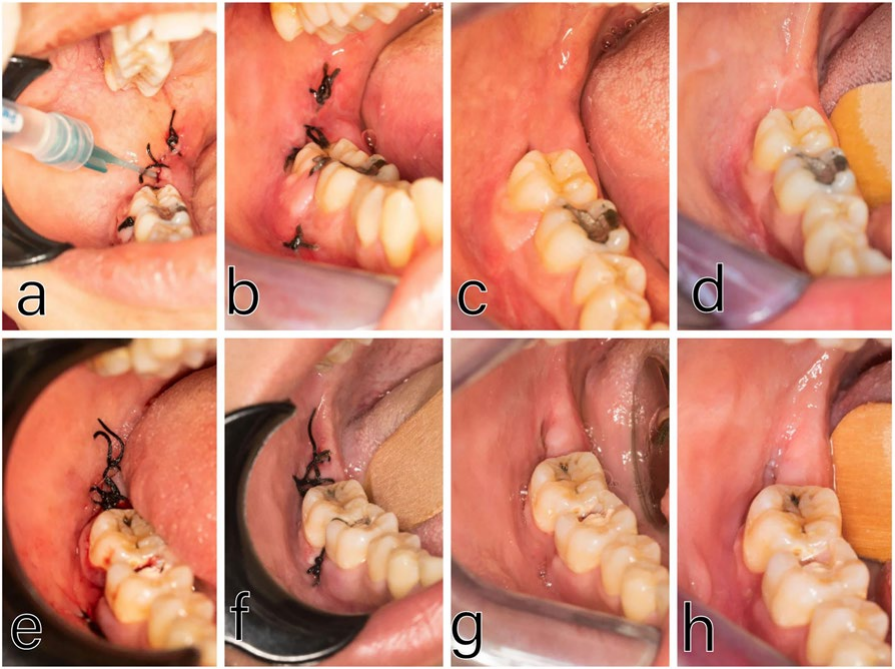
Above: A case of the Hyaluronic Acid group showing administration of HA (**a**), soft tissue healing at day 3 (**b**), day seven (**c**), and day fourteen (**d**). Below: A case of the control group showing suturing after surgery (**e**), Soft tissue healing at day 3 (**f**), day seven (**g**), and day fourteen (**h**)

**Fig. 3:**
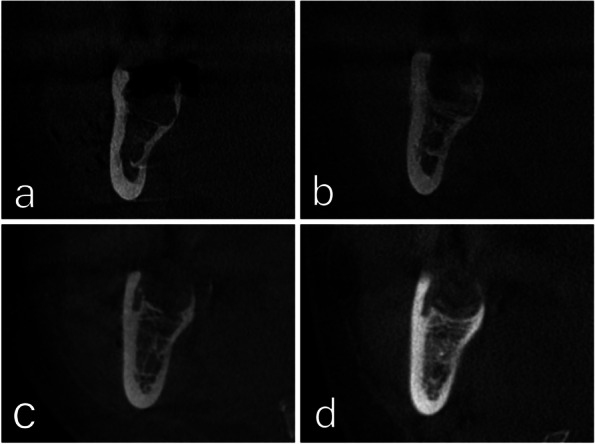
Comparison of bone density of study case (**A**, **B**) and control case (**C**, **D**) in immediate post-extraction (**A**, **C**) and after 2 months post extraction (**B**, **D**)

**Fig. 4:**
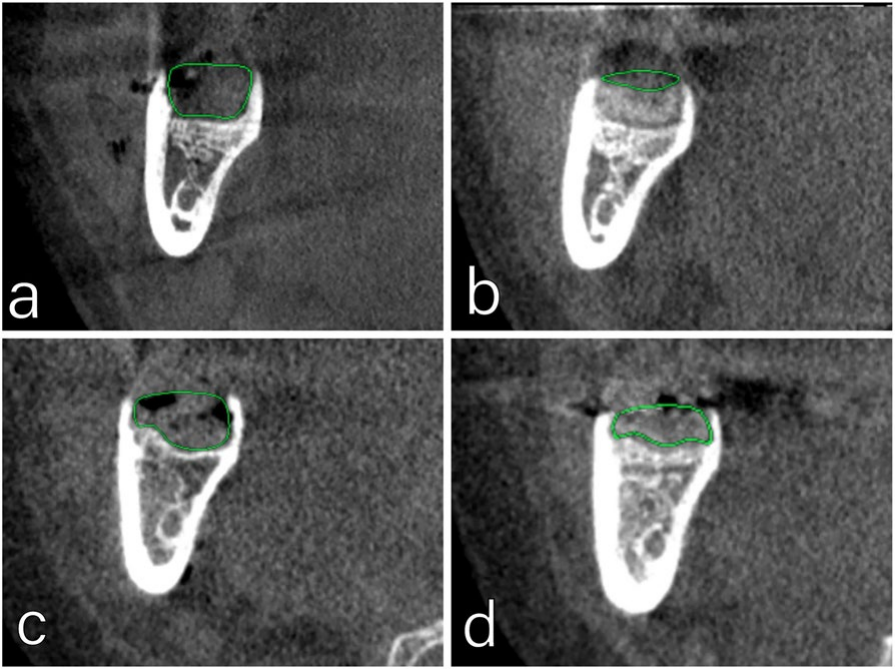
Comparison of the dimension of the socket of study case (**A**, **B**) and control case (**C**, **D**) in immediate post-extraction (**A**, **C**) and after 2 months post extraction (**B**, **D**)

The operator administered local anesthesia with Mepivacaine to block the inferior alveolar and lingual nerves, as well as providing infiltration anesthesia to the long buccal nerve. The surgical procedure, performed by the same operator for all cases, involved making a triangular incision with a No. 15 scalpel blade and raising a full mucoperiosteal flap. A round bur attached to a W&H surgical low-speed handpiece was then used to complete the osteotomy. Following proper osteotomy and bone separation, the tooth was extracted using an elevator. After extraction, the jagged bone edges were smoothed using a bone file. The socket was then cleaned and washed with saline solution, and finally, the wound was closed with sutures. No HA Group received simple interrupted sutures to close the wound. The extraction site of the HA Group was filled with half of the syringe of HA gel using a sterile disposable plastic needle (Fig. 1). Then, the wound was sutured with simple interrupted sutures. The remaining half of the HA gel was applied in the socket through small gaps between knots to ensure that the HA gel filled the socket. Following the surgical operation, patients were advised to keep a pressure pack between their arches for around half an hour (Figs. 1, 2, 3 and 4).

### Post-surgical phase

#### Postoperative instructions

Patients were instructed to use cold fomentation on the day of the operation, switching to hot fomentation from the second day until the end of the week. They were also advised to follow a soft diet. Sutures were removed seven days after the procedure.

- Patients were to adhere to the prescribed medication regimen, which included taking 875 mg of amoxicillin combined with 125 mg of clavulanic acid every 12 h for five days.
- Non-steroidal anti-inflammatory medicines every eight hours for four days.
- Chymotrypsin Trypsin at a dosage of 300 E.A.U every eight hours for five days.

#### Clinical and radiographic evaluation

- The soft tissue healing of cases was assessed on the 3rd, 7th, and 14th postextraction days. The same investigator adopted the healing index by Landry et al. [20], which included five scoring levels for each of the four parameters (Table 1) and recorded the relevant response.
- Each patient underwent radiological examination for bone healing using Cone Beam Computed Tomography (CBCT) at two postoperative intervals: immediately (baseline) and 60 days after surgery. The CBCT scans were acquired with a J. Morita R100 (J. Morita MFG. Corp., Kyoto, Japan). The images obtained were analyzed for bone density and socket dimensional changes. One examiner, who was blinded to which sockets had received hyaluronic acid, assessed the parameters.
- Radiographic bone density changes were evaluated by comparing baseline CBCT scans with scans obtained two months after the operation. Information was exported in DICOM format and transferred to be demonstrated by the OnDemand 3DTMI software program (OnDemand 3DTM Goddard Way, Suite 250 Irvine, CA 92618 USA. The density value was assessed in three distinct sections of the socket: the mesial, middle, and distal cuts. These sections were represented as squares of equal size and positioned at the center of the socket, aligned along the central line of the socket in the coronal view of the final scans. The density values from these sections were averaged to provide an overall measurement, with the values recorded numerically.

**Table 1:** Healing of soft tissue Index by Landry et al. (1985) [20]

Grade	Manifestation
Grade 1: Very poor	The tissue color should be equal to or over fifty percent of gingiva red Response to palpation: hemorrhage Suppuration: present Granulation tissue: present
Grade 2: Poor	The tissue color should be equal to or over fifty percent gingiva red Response to palpation: hemorrhage Suppuration: none Granulation tissue: present
Grade 3: Good	Tissue color should be greater than or equal to twenty-five percent but under fifty percent of gingiva red Granulation tissue: none Response to palpation: no hemorrhage Suppuration: none
Grade 4: Very good	Tissue color less than twenty-five percent gingiva red Response to palpation: no hemorrhage Suppuration: none Granulation tissue: none
Grade 5: Excellent	Response to palpation: no hemorrhage Tissue color: all tissue is pink & healthy Suppuration: none Granulation tissue: none

#### Socket length

A measurement was taken of the distance between the apex and the cervical limit of the bone in the most central slice of the radiographs immediately after the operation. This measurement served as a baseline for comparison with the sixty-day tomography, to reflect the amount of bone deposition from deepest point of the socket which reflect the bone deposition which starts from downward to upward.

#### Ethical consideration

The research protocol received approval (22 January 2023) from the Ethics Committee of Alexandria University Faculty of Dentistry (IRB No. 001056 – IORG 0008839) before any research-related activity commenced. This statement confirms that all research activities, including human subjects, adhered to the Declaration of Helsinki and other ethical norms established by the Research Ethics Committee of Alexandria University Faculty of Dentistry. The trial is retrospectively registered (30 July 2024) at the Pan African Clinical Trial Registry with the identification number for the registry PACTR202407576478340.

*Outcomes: The primary outcome* is Soft tissue healing after-extraction: three, seven and fourteen days postoperative. *Secondary outcomes* are bone density (Immediate, and two months postoperative); and socket length (Immediate, and two months postoperative).

#### Statistical analysis

- Statistical Package for Social Science (SPSS) software (version 25) [21] was used for data analysis. Shapiro–Wilk normality test proved the lack of normal distribution of the data, so, non-parametric statistics were utilized [22]. Mann-Whitney U [23], and Wilcoxon Signed Ranks tests [24] and Z-test for comparison of independent proportions [25] were used. During sample size calculation, beta error accepted up to 20% with a power of study of 80%. An alpha level was set to 5% with a significance level of 95%. Statistical significance was tested at *p*-value < 0.05 [26]

## Results

**Fig. 5:**
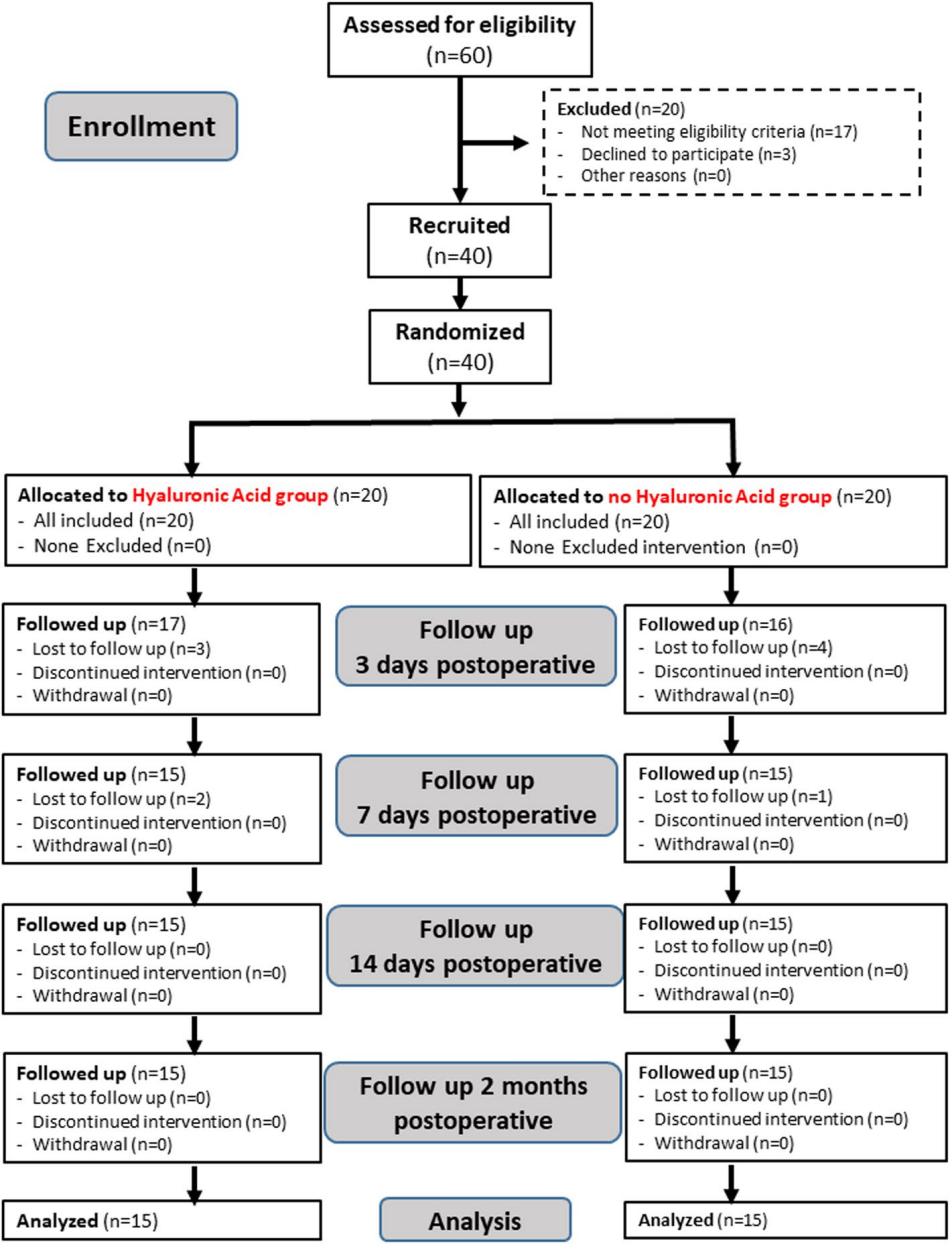
Consort flow diagram

Data are presented in median [25th—75th percentile (Inter-Quartile Range (IQR))]. Twenty patients were allocated in each intervention, only 15 patients in each group were completed the follow up (Fig. 5).

### Demographic data

The two studied groups were age and sex-matched (Table 2).

**Table 2:** Demographic data of the studied patients

Demographic data	Group		Test of significance *p-value*
	With HA (*n* = 15)	Without HA (*n* = 15)	
**Age (years)**			
- Min. – Max	21.00–36.00	22.00–35.00	
- Median	29.00	27.00	Z_(MW)_ = 1.394
- 95% CI of the median	27.00–34.00	24.00–32.00	*p* = .163 NS
- 25th Percentile – 75th Percentile	25.00–33.00	23.00–30.00	
**Sex**			
- Male (n = 10) (33.33%)	5 (33.33%)	5 (33.33%)	χ^2^_(df=1)_ = 0.000
- Female (n = 20) (66.67%)	10 (66.67%)	10 (66.67%)	*p* = 1.000 NS

*n* Number of patients

*Min–Max* Minimum – Maximum

*CI* Confidence interval

*Z* Z test of Mann–Whitney U test

*Df* degree of freedom

*NS* Statistically not significant (*p* ≥ 05)

χ^2^ = Pearson Chi-Square

^*^: Statistically significant (*p* < .05)

### Soft tissue healing (STH) index (Table 3)

**Table 3:** Soft tissue healing Index according to Landry et al. (1985) [20] in the studied groups

Wound Healing Index	Group		Test of significance *p-value*
	HA (*n* = 15)	No HA (*n* = 15)	
**Three days postoperative**			
- Very Poor (*n* = 11) (36.67%)	2 (13.33%)	9 (60.00%)	**Z = 2.652, ** ***p*** = .008*
- Poor (*n* = 17) (56.67%)	11 (73.34%)	6 (40.00%)	Z = 1.842, *p* = 065 NS
- Good (*n* = 2) (6.67%)	2 (13.33%)	0 (0.00%)	Z = 1.463, *p* = .144 NS
- Very Good (*n* = 0) (0.00%)	0 (0.00%)	0 (0.00%)	NA
- Excellent (*n* = 0) (0.00%)	0 (0.00%)	0 0.00%)	NA
**Seven days postoperative**			
- Very Poor (*n* = 0) (0.00%)	0 (0.00%)	0 (0.00%)	NA
- Poor (*n* = 13) (43.33%)	2 (13.33%)	11 (73.33%)	**Z = 3.315, ** ***p*** ** < .001**
- Good (*n* = 13) (43.33%)	9 (60.00%)	4 (26.67%)	Z = 1.842, *p* = .065 NS
- Very Good (*n* = 4) (13.34%)	4 (26.67%)	0 (0.00%)	**Z = 2.148, ** ***p*** = .031*
- Excellent (*n* = 0) (0.00%)	0 (0.00%)	0 (0.00%)	NA
**Fourteen days postoperative**			
- Very Poor ((*n* = 0) (0.00%)	0 (0.00%)	0 (0.00%)	NA
- Poor (*n* = 2) (6.67%)	0 (0.00%)	2 (13.33%)	Z = 1.463, *p* = 144 NS
- Good (*n* = 9) (30.00%)	0 (0.00%)	9 (60.00%)	Z = 3.585, *p* < .001
- Very Good (*n* = 11) (36.67%)	7 (46.67%)	4 (26.67%)	Z = 1.136, *p* = .254 NS
- Excellent (*n* = 8) (26.67%)	8 (53.33%)	0 (0.00%)	**Z = 3.302, ** ***p*** < .001*
**When adding Very good and Excellent**	15 (100.00)	4 (26.67%)	**Z = 4.167, ** ***p*** < .001*

*n* Number of patients

*Z* Z for comparison of two independent proportions

*NS* Not statistically significant (*p* ≥ .05)

^*^ Statistically significant (*p* < .05)

#### Three days postoperative

Out of the HA Group there were two (13.33%) with very poor STH, 11 (73.34%) with poor STH Index, and 2 (13.33%) with good STH Index. In the No HA Group, 9 (60.00%) with very poor STH Index, and 6 (40.00%) with poor STH Index. Patients with very poor STH Index were statistically significantly more in No HA three days postoperatively (*p* = 0.008). *Seven days postoperative:* In the HA Group, 2 (13.33%) with poor STH Index, 9 (60.00%) with good STH, and 4 (26.67%) with very good STH Index. In the No HA Group, 11 (73.33%) had a poor STH Index, and 4 (26.67%) had a good STH Index. Patients with a very good STH Index were statistically significantly more in the HA group than the No HA group (*p* = 0.031). *Fourteen days postoperative:* In the HA Group, 7 (46.67%) had a very good STH Index, and 8 (53.33%) had an excellent STH Index. In the No HA Group, 2 (13.33%) had a poor STH, 9 (60.00%) had a good STH Index, and 4 (26.67%) had a very good STH. Patients with Excellent (*p* < 0.001) or when combining very good to excellent (*p* < 0.001) were statistically significantly more in the HA group (Table 3).

### Bone density (HU)

*Immediate postoperative:* The median Bone Density was −83.70 HU, in the HA Group, while, in the No HA it was −126.77 HU with no statistically significant difference in Bone Density between the two studied groups (*p* = 0.319). *Two months Postoperative:* The Bone Density in the HA Group (median 125.60 HU) was statistically significantly higher than in the No HA Group (median 5.20 HU) (*p* < 0.001). Bone Density statistically significantly increased two months postoperative compared with immediate postoperative in the HA Group (*p* = 0.001) and in the no HA group (*p* = 0.001). Bone Density Percentage Change in the HA Group (median 239.20%) was statistically significantly higher than in the No HA Group (median 111.90%) (*p* < 0.001) (Table 4).

**Table 4:** Bone density of the studied groups

Bone density (HU)	Group		Test of significance *p-value*
	HA (*n* = 15)	No HA (*n* = 15)	
**Immediate postoperative**			
- Min. – Max	−185.70—−40.70	−285.78—54.80	
- Median	−83.70	−126.77	Z_(MW)_ = 0.996
- 95% CI of the median	−116.30—−52.40	−160.70—−48.70	*p* = .319 NS
- 25th Percentile – 75th Percentile	−122.60—−52.40	−184.40—−48.70	
**Two months postoperative**			
- Min. – Max	22.10—302.70	−160.40—176.60	
- Median	125.60	5.20	Z_(MW)_ = 3.754
- 95% CI of the median	92.80—205.30	−63.70—66.20	*p* < .001
- 25th Percentile – 75th Percentile	90.20—205.30	−84.34—66.20	
**Test of significance**	Z_(WSR)_ = 3.408	Z_(WSR)_ = 3.408	
***p-value***	*p* = .001*	*p* = .001*	
**Percentage change (%)**			
- Min. – Max	145.90—350.10	51.00–186.30	
- Median	239.20	111.90	Z_(MW)_ = 4.334
- 95% CI of the median	179.40–275.90	89.50—121.80	*p* < .001*
- 25th Percentile – 75th Percentile	178.70–275.90	78.40–121.80	

*n* Number of patients

*Min–Max* Minimum – Maximum

*CI* Confidence interval

*MW* Mann–Whitney U test

*WSR* Wilcoxon Signed Ranks Test

*NS* Statistically not significant (*p* ≥ 05)

^*^: Statistically significant (*p* < .05)

### Socket Length (mm) (Table 5)

*Immediate postoperative:* There was no statistically significant difference in the Socket length between the HA Group (median 6.14 mm) and No HA Group (median 6.17 mm) (*p* = 0.787). *Two months Postoperative:* Also, there was no statistically significant difference in the Socket length between the HA Group (median 5.12 mm) and No HA Group (median 5.65 mm) (*p* = 0.085). However, the percentage decrease in the socket length in the HA group (median −21.71%) was statistically significantly more than in the No HA group (median −12.66%) (*p* = 0.003). The socket length statistically significantly decreased two months postoperative compared with immediate postoperative in the HA Group and the No HA Group (*p* = 0.001 and *p* = 0.001, respectively) (Table 5).

**Table 5:** Socket length of the studied groups

Socket Length (mm)	Group		Test of significance *p-value*
	With HA (*n* = 15)	Without HA (*n* = 15)	
**Immediate postoperative**			
- Min. – Max	5.44–8.15	4.37–15.72	
- Median	6.14	6.17	Z_(MW)_ = 0.270
- 95% CI of the median	5.98–6.51	5.99–8.32	*p* = .787 NS
- 25th Percentile – 75th Percentile	5.89–6.51	5.53–8.32	
**Two months postoperative**			
- Min. – Max	3.85–5.93	3.59–13.62	
- Median	5.12	5.65	Z_(MW)_ = 1.722
- 95% CI of the median	4.91–5.56	4.98–7.48	*p* = .085 NS
- 25th Percentile – 75th Percentile	4.12–5.47	4.87–7.48	
**Test of significance**	Z_(WSR)_ = 3.408	Z_(WSR)_ = 3.408	
***p-value***	*p* = .001*	*p* = .001*	
**Percentage change (%)**			
- Min. – Max	−35.62—−11.89	−34.25—−3.40	
- Median	−21.71	−12.66	Z_(MW)_ = 2.966
- 95% CI of the median	−26.99—−14.54	−15.10—−7.53	*p* = .003*
- 25th Percentile – 75th Percentile	−27.24—−14.54	−15.23—−7.53	

*n* Number of patients

*Min–Max* Minimum – Maximum

*CI* Confidence interval

*MW* Mann–Whitney U test

*WSR* Wilcoxon Signed Ranks Test

*NS* Statistically not significant (*p* ≥ 05)

^*^: Statistically significant (*p* < .05)

## Discussion

Hyaluronic acid is a biopolymer that occurs naturally. It is a prominent extracellular matrix constituent in virtually all mammalian tissues and fluids [27]. It facilitates the movement, growth, and gradual specialization of mesenchymal cells. Consequently, it has a crucial function in the process of tissue regeneration and repair. HA stimulates cell proliferation, migration, and angiogenesis in connective tissues, supports the proliferation of basal keratinocytes, and reduces scar tissue formation [28–30].

In the present study, age of all the included patients ranged between 21–36 years, females represented 66.67% of them. This is in agreement with Patel et al. (2017) [31] who found that there was a high incidence of mesioangular lower third molar impaction (33.97%), with (48.33%) of it in the age group 15–30 years, and with a female predominance (63.44%).

The findings of our study indicated significant improvements in soft tissue healing. Specifically, the HA group demonstrated greater healing on the 3rd, 7th, and 14th days after the extraction than the control group. Significant variations were seen across groups at three, seven, and fourteen days, (*p* = 0.006, *p* = 0.001, and *p* < 0.001, respectively). This may be due to the anti-inflammatory properties of hyaluronic acid and its ability to promote angiogenesis.

Ruggiero et al. (2024) [32], examined the impact of HA on enhancing post-extraction socket healing in patients. The results indicated that the application of HA may play a significant role in improving post-extractive wound healing in the first 2 weeks post-surgery. A statistically significant difference was observed at both D7 and D14, with 97.2% (n = 35) of sites in the HA-treated group achieving excellent healing by D14, compared to 72.2% (*n* = 26) in the control group. This suggests that untreated sites may experience delayed complete healing, consistent with the current understanding of wound healing in diabetic patients.

In contrast, Guazzo et al. (2018) [33], studied the use of amino acid and sodium hyaluronate gel after third molar extraction and found no significant difference in post-extraction healing between the treated and control groups.

According to Mostafa et al. [34], their results showed significant variation in the healing index among the sockets cured with hyaluronic acid gel and the untreated sockets in terms of soft tissue healing. Specifically, 50% of the sockets in the hyaluronic acid gel group showed excellent healing in the following ten days, while just 20% in the control group showed excellent healing. This is due to the capacity of hyaluronic acid to stimulate the production of inflammatory cytokines like TNF-α and IL-1β, which in turn promote angiogenesis, and activate keratinocytes and fibroblasts in the healing process.

Consistent with Onesti et al. [35], the research examined the impact of hyaluronic acid on wound closure. The researchers determined that HA enhances the speed at which wounds heal and the re-epithelialization process.

In line with our study, Gocmen et al. [36] demonstrated that 0.8 percent hyaluronic acid exhibited an anti-inflammatory impact and significantly improved wound healing after operation. In addition to our research, Gocmen et al. [36] discovered that HA exhibits anti-inflammatory effects and promotes angiogenesis one week following the extraction. These results align with the findings of Ibraheem et al. [16], who demonstrated a significant impact of HA on the healing of wounds one week after extraction.

Regarding bone density, our results revealed that the HA group had statistically significantly higher bone density compared with the No HA Group (*p* < 0.001), although there was no statistically significant difference between the two studied groups immediately postoperative (*p* = 0.319),

The intragroup comparison demonstrated that there was a statistically significant increase in bone density two months postoperative compared to the immediate postoperative in the HA Group and the No HA Group (*p* = 0.001 and *p* = 0.001 respectively). However, the bone density percentage change revealed a higher increase in the HA Group compared with the No HA Group (*p* < 0.001).

Alcantara et al. (2018) [37] found that sockets treated with one percent HA gel had a greater percentage of bone formation (58.17%) and fractal dimension values (1.098) compared to the control group (48.97% and 1.074, respectively) throughout the thirty-day postoperative period (*p* < 0.05).

The present study findings showed that the Socket length had no statistically significant difference between the HA Group compared with the No HA Group immediately and after two months of extraction, (*p* = 0.787 and *p* = 0.087, respectively). The intragroup comparison demonstrated that there was a statistically significant decrease in Socket length two months postoperative compared to the immediate postoperative in the HA Group and the No HA Group (*p* = 0.001 and *p* = 0.001 respectively). In addition, the Socket length percentage change revealed a higher decrease in the HA Group compared with the No HA Group (*p* = 0.003). Also, (2018) [37] mentioned that, after 30 days, there was a high rate of bone formation in human lower first premolar sockets filled with HA.

Kim et al. (2016) [38], investigated HA's effect on wound healing and bone formation in extraction sockets with chronic pathology in dogs and observed promising results, indicating enhanced healing and bone formation.

In contrast, Mostafa et al. [34], demonstrated variations in socket lengths among the control group and the group treated with hyaluronic acid gel at different follow-up intervals. Immediately, on days five and ten, no statistically significant distinctions were observed between the control group and the hyaluronic acid gel group regarding the reduction of socket length and the occurrence of complications following surgery. However, the findings demonstrated that hyaluronic acid improves and accelerates the healing ability.

## Conclusion

Our research indicates that using an intra-socket HA gel is a viable way to improve and accelerate soft tissue healing following surgical extraction of the mandibular 3rd molar and better bone quality that permits early restoration of prosthetics, particularly in the case of dental implants.

## Recommendations

Based on the clinical results of this study, HA appears to be effective in enhancing both the timing and quality of post-extraction wound healing. However, additional clinical research and histological investigations are needed to further validate these findings.

## Limitations

The methodology precluded bone biopsy, which restricts the study and prevents histological analysis. It may be possible to expedite alveolar bone growth with HA by employing histological evaluation in human clinical investigations in the future. Also, the gel consistency of HA needs careful manipulation not to escape from the extraction socket.

## Declarations

## Data Availability

The data that support the findings of this study are available from the corresponding author, Omnia Saad Elkady, upon reasonable request.
